# Linking social preferences and ocean acidification impacts in mussel aquaculture

**DOI:** 10.1038/s41598-019-41104-5

**Published:** 2019-03-18

**Authors:** Valeska A. San Martin, Stefan Gelcich, Felipe Vásquez Lavín, Roberto D. Ponce Oliva, José I. Hernández, Nelson A. Lagos, Silvana N. R. Birchenough, Cristian A. Vargas

**Affiliations:** 1 Department of Aquatic Systems, Faculty of Environmental Sciences, Universidad de Concepcion, Concepcion, Chile; 2 Centre for the Study of Multiple-Drivers on Marine Socio-Ecological Systems (MUSELS), Universidad de Concepción, Concepción, Chile; 3 Centre of Applied Ecology and Sustainability, Department of Ecology, Pontificia Universidad Católica de Chile, Santiago, Chile; 4 School of Economics and Business, Universidad del Desarrollo, Concepcion, Chile; 5 Centro de Investigación e Innovación para el Cambio Climático (CiiCC), Facultad de Ciencias, Universidad Santo Tomás, Santiago, Chile; 6 Marine Climate Change Centre (MC3), Cefas, Lowestoft Laboratory, Suffolk, United Kingdom; 7 Millennium Institute of Oceanography (IMO), Universidad de Concepcion, Concepcion, Chile

## Abstract

Ocean Acidification (OA) has become one of the most studied global stressors in marine science during the last fifteen years. Despite the variety of studies on the biological effects of OA with marine commercial species, estimations of these impacts over consumers’ preferences have not been studied in detail, compromising our ability to undertake an assessment of market and economic impacts resulting from OA at local scales. Here, we use a novel and interdisciplinary approach to fill this gap. We experimentally test the impact of OA on commercially relevant physical and nutritional attributes of mussels, and then we use economic discrete choice models to assess the marginal effects of these impacts over consumers’ preferences and wellbeing. Results showed that attributes, which were significantly affected by OA, are also those preferred by consumers. Consumers are willing to pay on average 52% less for mussels with evidences of OA and are willing to increase the price they pay to avoid negative changes in attributes due to OA. The interdisciplinary approach developed here, complements research conducted on OA by effectively informing how OA economic impacts can be analyzed under the lens of marginal changes in market price and consumer’ welfare. Thereby, linking global phenomena to consumers’ wellbeing, and shifting the focus of OA impacts to assess the effects of local vulnerabilities in a wider context of people and businesses.

## Introduction

Linking the effects of global environmental drivers over human wellbeing associated to food security is a key area of research across multiple disciplines^1^. Shellfish aquaculture one of the largest food production sectors globally (USD$ 19 billion^2^) could experience significant economic losses and social disruptions due to changing ocean conditions, namely OA^3,4^. Mussel production ranks third, within the category of shelled mollusks cultivated globally^5^, supporting a global aquaculture industry worth more than US$3.0 billion in 2015^6^. Studies addressing the effects of OA over mussels have focused either on biological^5^ or economic dimensions^4,7^ with the objective of estimating overall population sustainability^5^, revenue^4^ or vulnerability^7^ of the industry^8^. While these studies have been important to highlight the potential impacts of OA over gross production and revenues, they do not link biological impacts with social preferences and ultimately human wellbeing. The ability to link biological and nutritional impacts of OA to consumers’ preferences is key, as it will allows to connect global phenomena with local scale impacts, thereby providing place-based information which allows to estimate OA impacts over businesses and people’s wellbeing^9^.

Studies have reported effects of OA on mussel biological attributes such as survival, shell dissolution, calcification rates^4,10,11^, shell growth rates, ingestion, respiration^12–14^, and increased vulnerability to diseases and parasites^5,15,16^. These biological changes could directly affect commercial attributes associated to mussel quality, such as taste, appearance, and nutritional composition^17–19^. Studies have also assessed potential economic impacts of OA on shellfish production^3,8,20,21^, However, to date, research aimed at assessing the effects of OA over commercial attributes of mussels has not been addressed in detail, hindering opportunities to link impacts of OA over consumers’ preferences and wellbeing (Table S1). In order to fill this gap, we adopted an interdisciplinary approach. We first experimentally assessed the impacts of OA over different physical (i.e. shell color) and nutritional (i.e. vitamin B12, protein content, and fatty acid composition) attributes of mussels under controlled laboratory conditions. Secondly, we adopted an economic valuation approach (i.e. discrete choice experiments) to assess consumers’ preferences of these attributes, undertaken with in-person surveys. This approach helped to: (i) characterize consumers’ preferences, (ii) link the preferences to the biological impacts of OA, and (iii) estimate the effects of OA, as a global driver, over consumers’ wellbeing when purchasing mussels at local scales. We ground-truth our approach using the Chilean mussel aquaculture industry, which is one of the lead industries in mussel production worldwide^22,23^.

## Results and Discussion

**Figure 1:**
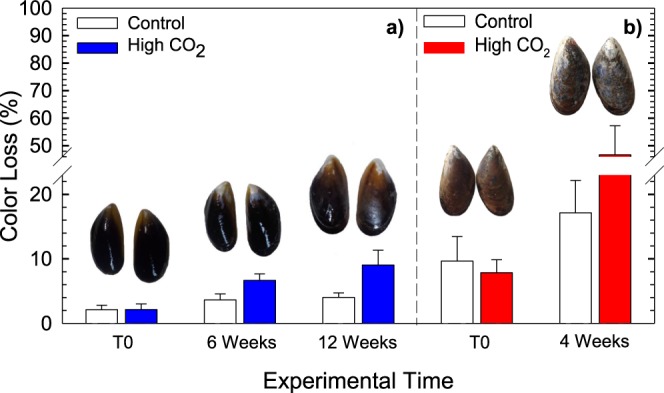
Summary of experimental results from changes observed over the physical attribute of mussels exposed to high *p*CO_2_ conditions. Mean ± standard deviation of color loss (%) in (**a**) juvenile mussels after 120 d (n = 30) and (**b**) adults mussels after 30 d (n = 10) exposed to high *p*CO_2_ and control *p*CO_2_ conditions. Significant statistical differences were found between mussels subjected to both treatments in juvenile (ANOVA, a priori comparison between treatments along time: F(2, 87) = 6.96; p = 0.002) and adults mussels (F(1, 18) = 91.88; p < 0.001).

A set of physical and nutritional attributes of mussels, which have been described as relevant in terms of commercial value^24^ and human wellbeing^25^ were experimentally tested to assess the effects of OA. Those attributes were classified into 1) appearance, which includes the physical attribute experimentally tested (shell color), and others identified as relevant for consumers and potentially affected by OA (shell size and meat color), and 2) nutritional characteristics, which include vitamin B12, protein content, and fatty acid composition (SFA, MUFA and PUFA) (Table 1, column 1, column 2, and column 3). Our results evidenced that physical attributes were significantly impaired under OA conditions. Figure 1 shows that as seawater pH declines from 7.9 to 7.6 (according to the RCP 8.5 scenario for 2100)^26^ on the total scale (i.e. CO_2_ increases from 400 to 1,000 ppm), the outer surfaces of shells deteriorated and its color was clearly lost in adults (≈50%) and juveniles (≈10%) (Table 1, column 4; Table S2). The deterioration in the outer layer (periostracum) of the mussel shell exposed to high *p*CO_2_ is similar to that found other studies^27–29^. For instance, whelk and oyster shells presented greater whitening as the pH becomes lower, resulting in a visually unattractive product from a marketing perspective^30^. Furthermore, our study is consistent with results observed by Osores *et al*.^31^, which links the trade-offs between shell carbonates precipitation and periostracum thickness of *M. chilensis* when exposed to low Ω_aragonite_ corrosive estuarine waters. It is well known that outermost layer of the periostracum provides most of mussel shell coloration^32^, thus these external changes may be attributed to acidification-induced reduction in shell thickness due to increased abrasion^33^ and/or alteration in the concentration of organic compounds^34^ of the shell periostracum.

**Table 1:** Summary of evaluations conducted under relevant physical and nutritional attributes of the commercial and human welfare in mussels affected by ocean acidification.

Category	Attributes Measured	Impact on market and/or wellbeing	OA experimental results.	Attributes’ social valuation (%)*	Attributes’ WTP**	WTP for 250 g of mussels quality loss***	Previous study	Previous study approach
Appearance	SHELL SIZE	impact on marketability, consumer election and buyer rejections	not tested	shell size: valued by 53,42% of respondents.	Shell size US$ 0.25	From Baseline product to shell size loss product: US$10.04 ->US$9.81	Adams *et al*., 2011^76^ Batzios *et al*., 2004^77^ Penney *et al*., 2007^30^	Growers perception (Slightly Economic) Consumer attitude (Social) Variability on appearance (Biological)
	MEAT COLOR	impact on marketability, consumer election and buyer rejections	not tested	meat color: valued by 57,90% of respondents.	Color of the meat US$ 1.22	From shell size loss product to color meat loss product: US$ 9.81 -> US$8.73		
	SHELL COLOR	impact on marketability, consumer election and buyer rejections	negative	not decolotated shell color: valued by 73,43% of respondents.	Color of the shell US$ 3.78	From color meat loss product to color shell loss product US$ 8.73 -> US$5.75		
Nutritional Characteristics	OMEGA-3	impact on marketability, consumer election and buyer rejections/ Vascular benefits, lower triglyerides, cardiac filling and myocardial efficiency, inflammation, thrombosis, and arrhythmia	negative	nutritional characteristics: valued by 61.65% of respondents.	nutritional characteristics US$ 1.39	From color shell loss product to nutritional characteristics loss product: US$ 5.75 -> US$4.79	Mozaffarian *et al*., 2011^41^ Grienke *et al*., 2014^25^	Benefits on human health (Biological) Effect on human health (Biological)
	EPA & DHA	impact on marketability, consumer election and buyer rejections/ reduced risk of cardiovascular events, diabetes mellitus, inhibiting growth of tumor cells, antiinflammatory activity, essential for fetal development	neutral				Kaur *et al*., 2011^40^ Swanson *et al*., 2012^39^	Benefits on human health (Biological) Benefits on human health (Biological)
	VIT B12	impact on marketability, consumer election and buyer rejections/ Essential for metabolism of fats and carbohydrates and the synthesis of proteins	negative				Huskisson *et al*., 2007^78^ Lund, 2013^79^	Importance in metabolism (Biological) Benefits in human health (Biological)
	PROTEIN	impact on marketability, consumer election and buyer rejection/ proteins are highly digestible and have a high biological value	negative				Tacon and Metian, 2013^80^	Human nutrition (Biological)

*Distribution of heterogeneity of preferences for mussel’s attributes, under normally distributed random coefficients. For instance, consumers showed preferences for OA-free shells (73.43%), the remaining 26,57% of respondents does not show preferences for this attribute.

**Marginal Willingness to Pay (MWTP) for an improvement in the selected attribute.

***The maximum Willingness to Pay (WTP) for 250 gr of mussel.

When evaluating consumers’ choices for mussels with different combinations of physical attributes, shell appearance was the most preferred attribute (Table 1, column 5). Our results indicated that more than 70% of the respondents preferred mussel shells that did not show evidence of OA impacts (i.e. not decolored). The relative importance of attributes in consumers’ wellbeing (i.e. shell color), can be expressed in term of the Marginal Willingness to Pay (MWTP). The MWTP for changes in shell color was US$3.78, meaning that consumers are willing to pay up to US$ 3.78 to avoid a negative change, due to OA, on shell color per 250 g of mussels (Table 1, column 6; Table S3). On the other hand, 55% of the respondents also preferred large shell size and yellow meat color, attributes not tested experimentally in this study, but which have shown to be susceptible to OA (Table 1, column 5). Shell appearance as a quality attribute has been identified as key in consumers’ decisions, influencing purchase^35^ and market price^36^. In this study, we have shown how OA is coupled to consumers’ wellbeing, for a set of appearance attributes (shell size, meat color, and shell color), jeopardizing local and national scale mussel market price. Our results showed that the maximum Willingness to Pay (WTP) decreases 57.2% from a baseline of a non-acidified product (USD$ 10.04), to a product with acidified appearance (USD$ 5.75) (Table 1, column 7). Thus, the variation of these attributes considered under future climate change scenarios could have serious implications to the mussel farming industry with impacts on the economy and human welfare.

**Figure 2:**
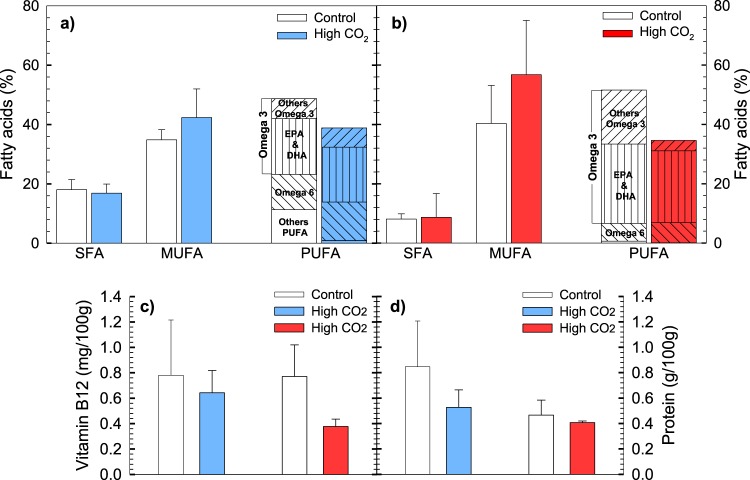
Summary of changes in nutritional attributes of mussels exposed to high *p*CO_2_ conditions. Mean (±S.D.), (**a**,**b**) fatty acid composition, and (**c**) vitamin B12 content in juvenile and adult and (**d**) Protein content, in both juvenile and adult mussels. Juvenile and adult individuals were exposed during 120 and 30 d to both treatments, respectively.

In our study the second most preferred attribute for consumers were the nutritional characteristics sush as Polyunsaturated Fatty Acids (PUFA), vitamin B12 and protein, which are preferred by 61% of respondents (Table 1, column 5). Research has shown that the consumption of mussels has been recommended for presenting important nutritional attributes^18,37^, which have been linked with positive effects on human health^38–41^ (Table 1, column 3). As expected, results showed that the mussel’s tissue was characterized by a high proportion of unsaturated fatty acids. The highest fatty acid proportion corresponds to monounsaturated (MUFA) and polyunsaturated fatty acids (PUFA) (Fig. 2a,b). Our results evidenced a significant impact of OA on the fatty acid composition. The PUFA content was significantly reduced under high *p*CO_2_ conditions for juvenile mussels (one-way ANOVA, F(1.12) = 4.83; p = 0.048) (Fig. 2a). Adults showed the same decreasing pattern in PUFA, although the comparisons did not show statistical significance (Fig. 2b). The PUFA content in this species is mostly contributed by Omega-3, with a major proportion of eicosapentaenoic acid (C20:5n-3, EPA) than docosahexaenocic acid (C22:6n-3, DHA) for both juvenile and adult mussels (Fig. 2a,b, respectively). The decrease in PUFA is mainly associated to stearidonic acid (C18:4n-3), α-linolenic acid (C18:3n-3), and docosapentaenoic acid (C22:5n-3) (Table S4). Importantly, analyses evidenced a lower protein content and Vitamin B12 in mussels exposed to high *p*CO_2_ for both juveniles and adults (Fig. 2c,d). The decrease in mussel nutritional quality upon high *p*CO_2_ conditions has been a major feature observed for other species. For instance, Valles-Regino *et al*.^42^ observed significant changes in fatty acid composition in whelks (i.e. reduction in Omega 3 PUFAs), and a reduction in the protein content of mussels^43^, oysters^44^, and whelks^45^ has been also observed upon high *p*CO_2_ conditions. Therefore, these changes can be attributed to a decrease in the ability to maintain lipid homeostasis^44,46^, a reduction in the abundance of proteins associated to the desaturation and elongation of fatty acids^47,48^ interfering in its synthesis^49^. Therefore, our results evidenced that stressful low pH/high *p*CO_2_ conditions might trigger significant changes in the physiology, metabolism and/or fatty acid storage of mussels.

Our survey results, which presented consumers with the nutritional importance of mussels compared to other food items and to the reductions in nutritional value associated to OA, showed that the MWTP for nutritional characteristics is US$ 1.39, meaning that consumers were willing to pay up to US$ 1.39 to avoid a negative change on nutritional quality per 250 g of mussels (Table 1, column 6). Maximum WTP decreases 16.6% from USD$ 5.75 for a product with acidified appearance to USD$ 4.79 for a product with also has reduced nutritional value (PUFA and vitamin B12 characteristics). Overall, the impacts of OA over mussels commercial attributes reduces consumers maximum WTP in 52% from USD$ 10.04 (baseline product- not acidified) to USD$ 4.79 (fully acidified product) (Table 1, column 7). This change is computed as follows: the maximum WTP will change from a not acidified product, to a product with change in the shell size in USD$ 0.23 (USD$ 10.04–USD$ 9.81). Then, the maximum WTP will change in USD$ 1.08 (USD$ 9.81–USD$ 8.73), from changing from a product affected by OA only in their shell size, to product in which also change the meat color. In general, the maximum WTP will change in USD$ 5.25 (USD$ 10.04–USD$ 4.79) for changing a not acidified product (baseline) to a fully acidified product (with changes in all the attributes).

Consumer responses revealed significant MWTP values for avoiding a decrease in quality of the attributes critically affected by the levels of OA predicted for the year 2100 [RCP8.5 scenario]^26,50^. These results did reflect the behavior of current consumer’s faced with future changes in mussel attributes and as such, did not consider the possibility of consumers lowering quality expectations in time. This possible shift in baselines cannot be assessed empirically as no market data exist for such a distant future^51^. However, data did show consumer attitudes were consistent in stating that appearance and nutritional levels of mussels were the most important attributes influencing purchasing behavior. Under this scenario, results suggest that OA will have real economic consequences at the individual level, which can have important consequences, not only by harming human wellbeing but also by increasing the vulnerability of producers being more affected by OA.

Linking global phenomena such as OA to tangible local industries and people’s wellbeing is a prerequisite for quantifying and planning effective place-based adaptation strategies^8^. Existing assessments and projections of the effects of OA over shellfish aquaculture production systems have in general estimated economic impacts through extrapolating direct losses in production associated to the effect of OA over calcification, growth rate, and larvae survival^20,21^. While this information is important for raising awareness of the potential risks, advocating direct measures to reduce global drivers of OA and safeguarding the shellfish industry, one of the biggest drawbacks is the lack of provision of information to support local industries to plan and adapt to potential market changes associated to this global driver. Through integrating the combined effects of biological responses related to commercial attributes, and the expected impacts over consumers’ marginal willingness to pay for those attributes, this study has contributed to furthering our current understanding of the effects of OA over the mussel industry. The interdisciplinary approach developed in this study, complements previous research conducted on OA by effectively informing how OA economic impacts can be analyzed under the lens of marginal changes in market prices, thereby linking global phenomena to consumers’ wellbeing, shifting the focus to local vulnerabilities for both people and businesses.

## Methods

### Organism collection

Juvenile and adults of *Mytilus chilensis* (Shell length: 2.5 ± 0.5 cm and 7.4 ± 0.2 cm, respectively) were collected at 5 m depth during July and October 2015 from a mussel farming facility located in Vilupulli, Chiloé, southern Chile (42°35′35″S; 73°47′18″W). Environmental conditions in the area averaged between 10.0 to 12.4 °C; 28.2 to 33.5 PSU of salinity, and 7.9 to 8.3 pH units. Once the organisms were collected, these were immediately transported to the Marine Biology Laboratory at Dichato (Universidad de Concepción). Individuals were kept in filtered (0.1 μm) and aerated seawater at a temperature of ~11 °C, ~31 psu salinity, and 8.0 ± 0.1 pH units, which indeed represented the mean ambient conditions in the mussel farming area during an annual cycle. An acclimatization period of two weeks under mesocosm conditions, animals were fed daily with the commercial food phytogold-s (*Isochrysis sp*. and *Pavlova sp*.), at a mean concentration of 2.5 μg L^−1^ Chl *a*.

### CO_2_ manipulation and experimental conditions

Two plastic 280 L tanks were used as acidification units to generate seawater to two nominal levels of *p*CO_2_: 400 μatm (control treatment) simulated present day conditions corresponding to the average level in mussel farming area^13^ and 1000 μatm (CO_2_ treatment) simulated the future conditions based on RCP8.5 scenario which predicts pH change rates of −0.0018 pH units by year^26,50^. The study was set up at two different level of *p*CO_2_, Air/CO_2_ mixtures, using a bulk flow technique, where know flow of dry air and ultra-pure CO_2_ gas were supplied, via mass flow controller (MFC), and mixed before equilibration with seawater. During the experiments seawater pH (total scale, pH_T_) was monitored in each tank every 10 day, measuring potentiometrically in 25 mL cell thermostatted at 25 ± 0.1 °C for standardization, using a Metrohm 713 pH meter (input resistance >1013 Ohm, 0.1 mV sensitivity, and nominal resolution 0.001 pH units) using a glass combined double junction Ag/AgCl electrode (Methrom model 6.0219.100) calibrate with 8.089 Tris buffer 25ªC. following DOE potentiometric method^52^. pH values are reported on the total hydrogen ion scale. Temperature and salinity were measured using an Oakton SALT6+ handheld salinity meter with probe. Total alkalinity was measured 7 days using the open-cell titration method^53^, by using an automatic Alkalinity Tritator Model AS-ALK2 Apollo SciTech. The AS-ALK2 system is equipped with a combination pH electrode (8302BNUWP *Ross Ultra* pH/ATC *Triode*, Thermo Scientific, USA) connected to a pH meter (Orion star A211 pH meter, Thermo Scientific, USA). All samples were analyzed at 25 °C (±0.1 °C) with temperature regulation using a water-bath (Lab companion CW-05G). The accuracy was controlled against a certified reference material (CRM, supplied by Andrew Dickson, Scripps Institution of Oceanography, San Diego, USA) and the A_T_ repeatability averaged 2–3 μmol kg^−1^.

The pH, total alkalinity (AT), phosphate and dissolved silicate^54^ used to calculate the rest of the carbonate system parameters and the saturation stage of omega, aragonite and calcite using CO_2_SYS software^55^ set with Mehrbach solubility constants^56^ refitted by Dickson and Millero (1987)^57^, presented in Supplementary Information Table 5. Seawater in each experiment was replaced every two days with seawater that had been previously balanced.

### Experimental setup

After the acclimation period, sixty individuals were cleaned and separated in ten groups of 6 individuals in aquariums with 9 L of capacity, each treatment was replicated five times with total 30 individuals for control (11 °C and 400 ppm CO_2_) and 30 individuals for high *p*CO_2_ treatment (11 °C and 1000 ppm CO_2_).

Color change determinations were measured with weekly photographs of each specimen in each treatment. All images were processed using ImageJ software (v.1.45 s; NIH, Bethesda, MD, USA). The exposure time to the different treatments was 120 days for juvenile and 30 days for adult. The long period of exposure for juvenile allowed us to make sure to detect significant changes in shell color. However, for adult individuals the exposure time was shorter, since adult mussels considered in our study were at pre-spawning stage (50 mm shell length, Lagos *et al*.^58^). It is well known that after that size, adult mussels change their energy expenditure destined mainly to the reproduction phase^59^. Therefore, they were prone to spawning, which could result in potential changes in their nutritional composition, affecting our comparative analysis with juvenile individuals. The samples of mussel tissue were obtained by means of the extraction of the gonads and the adductor muscle, for individual juvenile (n = 7) and adults (n = 2). Fatty acid analysis was performed in the Institute of Pharmacology at the Universidad Austral de Chile in Valdivia, Chile. The fatty acid concentration was analyzed after extraction and methylation^60^ with a gas chromatograph Perkin Elmer sigma 300 equipped with a programmable temperature vaporizer-injector, a fused Omegawax 53 capillary column, and a flame ionization detector and vitamin B12 by HPLC technique. Relative quantities were expressed as percent of total fatty acids in each sample.

### Data analysis

The proportional data of physical (color loss) and nutritional attributes (fatty acids composition and PUFA) was arcsine transformed for posterior analysis. Due the repeated measures of loss of color of the mussels’ tissues, we used ANOVA model nesting individual mussels (random effect) along time and *p*CO_2_ treatment (fixes effects). The effects of exposure to high *p*CO_2_ in nutritional attributes (fatty acids composition, PUFA, protein and vitamin B12 content) were analyzed using a one-way ANOVA followed by a Tukey post hoc test.

### Social valuation of commercial attributes

Social preferences were modeled using a Random Utility Model^61,62^. As mentioned above, we carried out a simulated experiment of impacts with a projection towards the years 2070–2100, corresponding to the rate of change in the pH predicted by the most extreme scenario [RCP8.5 scenario] of atmospheric CO_2_^26,50^. Considering that no market data exist for such distant future, and following^51,63^, we rely on the choice experiment approach^64^, providing the opportunity to consumers to declare their preferences choosing between several alternatives that were differentiated by the combination of levels of attributes, mainly known to be affected by OA, mainly appearance and nutrition. Surveys, which include choice experiments, have been widely used to study consumer preferences for bundles of attributes in the literature^65–67^ and have gained important traction in food preference and environmental literature during the past decades^68–73^. (Table S6).

We developed a choice experiment survey which complied with ethical approval of both project Musels and Universidad del Desarrollo. As such, all interviewees were presented with an informed consent form, which had to be approved. The in-person interviews were conducted from October to December 2016 in two Chilean cities: Santiago (Chile’s capital) and Concepción (the second-largest city). We also conducted four focus groups (two each in Concepción and Santiago) to explore people’s reactions to specific aspects of the experiment and to identify wording problems or misleading sections in the survey. Then, we conducted 125 pilot surveys to field-test the design of the instrument (one pilot with 25 observations and two pilots with 50 observations each). We rely on a random sampling process based on the National Socioeconomic Household Survey (CASEN), using a probabilistic polietapic sampling design, in which we randomly select neighborhoods and blocks. Next, we systematically select the households to be interviewed. Here, we select one household in each block, starting in the northern corner. If there is no answer from that house, we skip the next four houses and try the fifth. The sampling process yielded a useful sample of 1,278 individuals, each of whom were presented with six decisions, with three alternatives for each choice. This yielded a final sample of 7,668 useful observations.

A choice example used in the survey is presented in Supplementary Information Table 7. Following a D-optimality experimental design^74^ consumer interviewed face six choice scenarios with three alternatives each. For the choice experiment a consumer was presented with three alternatives included the combination of different levels of appearance with and without OA (e.g. small size, large size; pale meat, yellow meat; decolorated shell, not decolorated shell color), nutritional characteristics (low nutritional composition, high nutritional composition), and prices. Using visual aids, we informed interviewees about the differences in nutritional characteristic by referring to components of the PUFA complex, vitamin B12 and proteins as key nutritional characteristics of mussels. We presented information through figures and visual aids, which compared these characteristics with other food items which had similar nutritional attributes and that are perceived as healthy by the Chilean population (i.e salmon, and mackerel).

The model was estimated using random parameters allowing capturing unobserved heterogeneity (Table S3). Other attributes were also evaluated but we focus on those related to OA in this discussion.

The welfare attain by individual r for choosing alternative i, in RUM approach is given by Equation(1).

1 $${V}_{ir}={\alpha }_{ir}{X}_{i}+{{\epsilon }}_{i}$$

where X is a vector of attributes of the alternatives, ${{\epsilon }}_{i}\,\,$is an stochastic component that allow us to estimate a probability model and $\,{\alpha }_{ir}=\alpha +\beta S+\sigma {\eta }_{r}$ is a set of parameters to be estimated that depend on observed individual characteristics S (age, sex, education, etc.) and unobserved characteristics ${\eta }_{r}$ that are stochastically distributes in the population, ${\eta }_{r}$ is random component (different for each coefficient). The full model is given by Equation(2).

2 $${V}_{ir}=(\alpha +\beta S+\sigma {\eta }_{r}){X}_{i}+{{\epsilon }}_{i}=\alpha {X}_{i}+\beta S{X}_{i}+\sigma {\eta }_{r}{X}_{i}+{{\epsilon }}_{i}$$

If ${{\epsilon }}_{i}\,\,$has an Extreme value distribution type I, then the RUM is estimated using a random parameter logit model in which the probability of choosing alternative i is given by Equation(3).

3 $${P}_{i}=\int \frac{{e}^{{V}_{i}}}{{\sum }_{j}^{J}{e}^{{V}_{j}}}f({\alpha }_{r})\partial {\alpha }_{r}$$

where J is the total number of alternatives included in the choice sets. The sociodemographic variables capture the observed heterogeneity among individuals, and the random parameter ${\eta }_{r}$ captures the unobserved heterogeneity. Both sources of heterogeneity are crucial to understand people’s preferences for mussels. Using the fitted model, it is possible to estimate willingness to pay for each attribute and level of the attribute. The method allows us to estimate the relevance of each attribute of the alternative, in this case attribute differentiated mussel products, in the welfare function for each individual. With those results it is possible to estimate the marginal willingness to pay for each attribute and eventually aggregate this value to the extension of the mussel market^75^.

### Ethics statement

Through the consent of the Ethics, Bioethics and Biosafety Committee of the Vice-Rector for Research and Development of the University of Conception, President: Dr. Andrea Rodríguez Tastets. Checked compliance with the ethical, bioethical and biosecurity norms and procedures established nationally and internationally for research in the field of environmental sciences, considering the study of hydro biological species and that includes manipulation of biological and chemical material and waste. Written informed consent was obtained from the respective institution in Concepcion, Chile, previously approved the ethic protocol from all subjects for this study.

### Permission statement

Once the ethics protocol of the Universidad de Concepción was approved in this study, the correct permits for the collection of commercial molluscs were obtained, which were donated by a mussel farming facility located in Vilupulli, Chiloé, southern Chile.

## Supplementary information

Supplementary Information

## Data Availability

Any data used in this paper can be obtained by contacting the corresponding author.
