# Supplementary material for: Linking social preferences and ocean acidification impacts in mussel aquaculture
Source: Sci Rep. 2019 Mar 18;9:4719. doi: 10.1038/s41598-019-41104-5 (PMC6423318; doi:10.1038/s41598-019-41104-5)
Supplement: Supplementary file 1 — Supplementary Information [file 41598_2019_41104_MOESM1_ESM.docx]

**Supplementary Information**

*Linking social preferences and ocean acidification impacts in mussel aquaculture*

Valeska A. San Martin^1,2^, Stefan Gelcich^2,3^, Felipe Vasquez^2,4^, Roberto Ponce^2,4^, Ignacio Hernández^2,4^, Nelson A. Lagos^2,5^, Silvana N.R. Birchenough^6^, and Cristian A. Vargas^1,2,7*^

**Supplementary Tables**

**Supplementary Table 1. Literature review on OA impact on market attributes.** Summary of OA and other exposure conditions considered under market attributes of the main marine commercial species.

| Category | Attributes | Group | Species | Study | Nº study | Exposure conditions |
| --- | --- | --- | --- | --- | --- | --- |
|  |  |  |  |  |  |  |
| Physiological | shell size | Mussel | *Mytilus galloprovincialis* | Michaeledis et al., 2005 | 23 | OA |
|  |  |  | *Mytilus edulis* | Berge et al., 2006 |  | OA |
|  |  |  | *Bathymodiolus brevior* | Tunnicliffe et al., 2009 |  | Acidic water condition |
|  |  |  | *Mytilus edulis* | Gazeau et al., 2010 |  | OA |
|  |  |  | *Bathymodiolus brevior* | Thomsen et al., 2010 |  | OA |
|  |  |  | *Mytilus edulis* | Thomsen and Melzner, 2010 |  | Moderate seawater acidification |
|  |  |  | *Mytilus edulis* | Bechmann et al., 2011 |  | OA |
|  |  |  | *Mytilus edulis* | Melzner et al., 2011 |  | OA |
|  |  |  | *Mytilus californianus* | Gaylord et al., 2011 |  | OA |
|  |  |  | *Mytilus trossulus* | Sunday et al., 2011 |  | OA |
|  |  |  | *Mytilus galloprovincialis* | Range et al., 2012 |  | OA |
|  |  |  | *Mytilus edulis* | Appelhans et al., 2012 |  | OA |
|  |  |  | *Mytilus edulis* | Hiebenthal et al., 2012 |  | Oa and Temperature |
|  |  |  | *Mytilus edulis* | Thomsen et al., 2013 |  | OA |
|  |  |  | *Mytilus edulis* | Hiebenthal et al., 2013 |  | OA and Temperature |
|  |  |  | *Mytilus edulis* | Mackenzie et al., 2014 |  | OA and Temperature |
|  |  |  | *Mytilus edulis* | Asplund et al., 2014 |  | OA |
|  |  |  | *Mytilus galloprovincialis and Mytilus californianus* | Frieder et al., 2014 |  | OA and Oxigen |
|  |  |  | *Mytilus Galloprovincialis* | Bressan et al., 2014 |  | OA |
|  |  |  | *Mytilus Galloprovincialis* | Kroeker et al., 2014 |  | OA and Temperature |
|  |  |  | *Mytilus Galloprovincialis* | Gazeau et al., 2014 |  | OA and Temperature |
|  |  |  | *Mytilus galloprovincialis and Mytilus californianus* | Waldbusser et al., 2015 |  | OA |
|  |  |  | *Mytilus edulis* | Keppel et al., 2015 |  | OA and Temperature |
|  |  | Oyster | *Crassostrea gigas* | Kurihara et al., 2007 | 12 | OA |
|  |  |  | *Crassostrea virginica* | Beniash et al., 2010 |  | OA |
|  |  |  | *Saccostrea glomerata* | Parker et al., 2011 |  | OA |
|  |  |  | *Crassostrea virginica* | Talmage and Gobler, 2011 |  | OA and Temperature |
|  |  |  | *Crassostrea gigas* | Barton et al., 2012 |  | OA |
|  |  |  | *Saccostrea glomerata* | Amaral et al., 2012 |  | Estuarine Acidification |
|  |  |  | *Crassostrea virginica* | Dickinson et al., 2012 |  | OA and Salinity |
|  |  |  | *Crassostrea gigas* | Waldbusser et al., 2013 |  | OA |
|  |  |  | *Ostrea lurida* | Hettinger et al., 2013 |  | OA |
|  |  |  | *Crassostrea gigas* | Barros et al., 2013 |  | OA |
|  |  |  | *Ostrea lurida* | Stanford et al., 2014 |  | OA |
|  |  |  | *Saccostrea glomerata* | parker et al., 2015 |  | OA |
|  |  |  | *Saccostrea glomerata* | Scanes et al., 2017 |  | OA |
|  |  | Clam | *Ruditapes decussatus* | Range et al., 2011 | 4 | OA |
|  |  |  | *Mercenaria mercenaria* | Talmage and Gobler, 2011 |  | OA and Temperature |
|  |  |  | *Chamelea gallina* | Range et al., 2013 |  | OA |
|  |  |  | *Chamelea gallina* | Bressan et al., 2014 |  | OA |
|  |  | Nail | *Concholepas concholepas* | Manríquez et al., 2013 | 2 | OA |
|  |  |  | *Littorina littorea* | Melatunan et al., 2013 |  | OA and Temperature |
|  |  | Scallop | *Argopecten irradians* | Talmage and Gobler, 2011 | 2 | OA and Temperature |
|  |  |  | *Argopecten irradians* | White et al., 2013 |  | OA |
|  | Meat color | Oyster | *Crassostrea gigas* | Cruz-Romero et al., 2007; 2008a; 2008b; 2004 | 7 | High pressure |
|  |  | Fish | *salmon* | Alfnes et al. (2006) |  | - |
|  |  | Snail | *Haliotis sp.* | Brown et al. (2008) |  | - |
|  |  |  | *Haliotis rufescens* | Briones-Labarca et al., 2012 |  | high hydrostatic pressure |
|  | Shell appearance | Mussel | *Mytilus chilensis* | Osores et al., 2017 | 4 | Differences habitats |
|  |  | Oyster | *Crassostrea gigas* | Cruz-Romero et al., 2007; 2008 |  | high pressure |
|  |  |  | *Pinctada fucata* | Welladsen et al., 2010 |  | OA |
| Organoleptic | Texture | Snail | *Haliotis sp.* | Brown et al., 2008 | 4 | - |
|  |  | Fish | *wild and farmed fish* | Borderías & Sánchez-Alonso, 2011 |  | - |
|  | Taste-sea scent | Shrimp | *Pandalus borealis* | Dupont et al., 2014 |  | OA |
|  |  | Oyster | *Crassostrea gigas* | Lemasson et al., 2017 |  | OA and Temperature |
| Nutritional composition | Nutritional composition | Mussel | *Mytilus galloprovincialis* | Orban et al., 2002 | 8 | Differents habitats |
|  |  |  | *Mytilus galloprovincialis* | Fuentes et al., 2009 |  | Differents habitats |
|  |  |  | *Mytilus galloprovincialis* | Pettersen et al., 2010 |  | Food |
|  |  |  | *Mytilus edulis* | Both et al., 2011 |  | Differents habitats |
|  |  |  | *Mytilus edulis* | Colombo et al., 2016 |  | Differents habitats |
|  |  | Oyster | *Crassostrea gigas* | Soudant et al., 1999 |  | Differents habitats |
|  |  |  | *Crassostrea gigas* | Timmins-Schiffman et al., 2014 |  | OA |
|  |  | Seafood | *mainly fish* | Lloret et al., 2016 |  | Sea warming and OA |
| Product | Product depth/ assortment | Mussel | *Mussels* | Batzios et al., 2003; 2004 | 2 | − |

**Supplementary Table 2:** **Colour Loss (%) of mussels.** Mean (± SD) of juvenile and adult mussel samples at the end of the experiment.

|  |  |  |  |  |
| --- | --- | --- | --- | --- |
|  |  |  | Color Loss (%) | |
| Stage | Exposure Time | Sample | Control | CO_2_ |
| Juvenile | T0 | 30 | 1.99 ± 1.98 | 2.04 ± 1.76 |
|  | 6 Weeks | 30 | 3.61 ± 2.25 | 6.69 ± 3.07 |
|  | 12 Weeks | 30 | 4.05 ± 3.23 | 9.06 ± 4.04 |
|  |  |  |  |  |
| Adults | T0 | 10 | 9.53 ± 3.80 | 7.78 ± 2.01 |
|  | 4 Weeks | 10 | 17.48 ± 5.0 | 47.62 ± 10.57 |

**Supplementary Table 3: Estimation results for the Mixed Logit Model.** The mean and standard deviation for random coefficients are summarized, with their respective standard errors (in parenthesis), while fixed coefficients are presented with their standard errors. Positive (negative) fixed coefficients means a supportive (discouraging) valuation of the attribute in average. Random coefficients must be interpreted in terms of mean and Standard deviation: a positive (negative) mean coefficient implies that more than (less than) 50% of people should support the attribute, subject to a statistically significant value of the attribute’s standard deviation.

| Attribute | | Coefficients | |  |
| --- | --- | --- | --- | --- |
| Random coefficients | | Mean | SD |  |
| Shell Size | | 0.058 | 0.499*** |  |
|  | | (0.051) | (0.066) |  |
| Color of the shell | | 0.832*** | 1.003*** |  |
|  | | (0.066) | (0.054) |  |
| Color of the meat | | 0.268*** | 0.994*** |  |
|  | | (0.061) | (0.056) |  |
| Nutritional quality | | 0.308*** | 0.762*** |  |
|  | | (0.055) | (0.055) |  |
| Fixed coefficients | | Coefficient |  |  |
| Price | | -0.362*** |  |  |
|  | | (0.052) |  |  |
|  |  |  |  |  |

Standard errors in parenthesis. Means and SDs are statistically significant at a 1% level except Shell Size whose mean is not statistically significant but the SD is, suggesting consumer heterogeneity. ^***^ indicates a p-value<0.001.

**Supplementary Table 4: Fatty acid composition of mussels.** Mean (± SD) total profile content of fatty acids of juvenile and adult mussel samples at the end of the experiment.

|  | Juvenile | |  | Adults | |
| --- | --- | --- | --- | --- | --- |
| Fatty acids | Control | CO_2_ |  | Control | CO_2_ |
| Saturated (SFA) |  |  |  |  |  |
| MeC12:0 | 0.76 ± 0.24 | 0.61 ± 0.28 |  | 0.03 ± 0.01 | 0.08 ± 0.02 |
| MeC14:0 | 0.18 ± 0.09 | 0.17 ± 0.20 |  | 0.07 ± 0.03 | 0.09 ± 0.01 |
| MeC15:0 | 0.10 ± 0.10 | 0.10 ± 0.06 |  | 0.005 ± 0.00 | 0.01 ± 0.00 |
| MeC16:0 | 11.14 ± 2.97 | 10.11 ± 1.72 |  | 5.86 ± 1.10 | 6.42 ± 1.25 |
| MeC17:0 | 0.33 ± 0.26 | 0.50 ± 0.28 |  | 0.11 ± 0.01 | 0.17 ± 0.02 |
| MeC18:0 | 3.27 ± .65 | 3.25 ± 0.96 |  | 0.65 ± 0.25 | 0.72 ± 0.18 |
| MeC20:0 | 0.58 ± 0.24 | 0.51 ± 0.11 |  | 0.52 ± 0.15 | 0.10 ± 0.02 |
| MeC22:0 | 0.16 ± 0.03 | 0.14 ± 0.05 |  | 0.05 ± 0.02 | 0.06 ± 0.01 |
| MeC24:0 | 1.14 ± 0.20 | 1.11 ± 0.34 |  | 0.76 ± 0.15 | 0.98 ± 0.12 |
| MeC26:0 | 0.39 ±0.22 | 0.34 ± 0.14 |  | 0.03 ± 0.01 | 0.06 ± 0.01 |
|  |  |  |  |  |  |
| Monounsatured(MUFA) |  |  |  |  |  |
| MeC14:1 | 0.08 ± 0.04 | 0.07 ± 0.02 |  | 0.01 ± 0.01 | 0.02 ± 0.00 |
| MeC16:1 | 11.73 ± 6.99 | 18.50 ± 8.83 |  | 34.70 ± 22.29 | 49.33 ± 6.22 |
| MeC17:1 | 0.40 ± 0.12 | 0.40 ± 0.18 |  | 0.06 ± 0.02 | 0.07 ± 0.01 |
| MeC18:1 c n-9 | 20.97 ± 9.69 | 21.68 ± 11.11 |  | 3.74 ± 0.48 | 6.00 ± 2.08 |
| MeC18:1 t n-9 | 1.20 ± 0.68 | 1.35 ± 0.99 |  | 0.79 ± 0.07 | 0.76 ± 0.21 |
| MeC20:1 n-9 | 0.45 ± 0.21 | 0.36 ± 0.22 |  | 1.03 ± 0.66 | 0.55 ± 0.15 |
| MeC24:1 | 0.01 ± 0.01 | 0.004 ± 0.00 |  | 0.004 ± 0.00 | 0.002 ± 0.00 |
|  |  |  |  |  |  |
| Plyunsaturated (PUFA) |  |  |  |  |  |
| MeC18:2 c n-6 | 8.04 ± 5.69 | 8.09 ± 5.68 |  | 1.87 ± 0.44 | 2.31 ± 1.80 |
| MeC18:2 t n-6 | 0.13 ± 0.08 | 0.08 ± 0.06 |  | 0.52 ± 0.74 | 0.02 ± 0.00 |
| MeC18:4 n-3 | 0.13 ± 0.13 | 0.07 ± 0.03 |  | 14.76 ± 20.80 | 0.08 ± 0.00 |
| MeC18:3 n-3 | 6.46 ± 2.55 | 6.05 ± 1.61 |  | 3.42 ± 0.51 | 3.23 ± 0.23 |
| MeC18:3 n-6 | 0.66 ± 0.47 | 0.60 ± 0.29 |  | 1.62 ± 2.27 | 1.81 ± 0.27 |
| MeC20:2 | 0.67 ± 0.14 | 0.63 ± 0.27 |  | 0.27 ± 0.03 | 0.38 ± 0.04 |
| MeC20:3 n-6 | 0.98 ± 0.25 | 0.98 ± 0.33 |  | 0.18 ± 0.03 | 0.32 ± 0.02 |
| MeC20:4 n-6 | 0.30 ± 0.08 | 1.12 ± 1.25 |  | 0.10 ± 0.05 | 0.06 ± 0.03 |
| MeC20:5 n-3 | 15.64 ± 2.27 | 13.94 ± 4.77 |  | 26.10 ± 6.81 | 23.39 ± 4.57 |
| MeC22:3 n-6 | 11.16 ± 12.05 | 0.79 ± 0.64 |  | 0.11 ± 0.12 | 0.05 ± 0.01 |
| MeC22:4 n-6 | 1.40 ± 0.52 | 1.13 ± 0.50 |  | 2.00 ± 0.77 | 1.90 ± 0.46 |
| MeC22:5 n-3 | 0.60 ± 0.22 | 0.38 ± 0.12 |  | 0.03 ± 0.02 | 0.17 ± 0.02 |
| MeC22:6 n-3 | 4.09 ± 1.08 | 3.73 ± 0.81 |  | 0.62 ± 0.50 | 0.86 ± 0.11 |
| SFA | 18.05 ± 3.41 | 16.85 ± 3.10 |  | 8.08 ± 1.79 | 8.69 ± 1.98 |
| MUFA | 34.85 ± 3.41 | 42.37 ± 9.64 |  | 40.32 ± 12.83 | 56.73 ± 18.31 |
| PUFA | 50.27 ± 5.02 | 37.58 ± 4.15 |  | 51.63 ± 7.73 | 34.58 ± 6.31 |
| Σω 3 | 26.92 ± 5.98 | 24.16 ± 5.28 |  | 44.93 ± 13.04 | 27.72 ± 12.32 |
| Σω 6 | 12.05 ± 2.98 | 12.56 ± 2.95 |  | 6.04 ± 0.91 | 6.79 ± 0.98 |

**Supplementary Table 5: Water conditions during 120-day experiment.** Mean (±SD) conditions of carbonate system parameters during experiment conducted with *Mytilus chilensis.* Total alkalinity (TA in μmol Kg^-1^), partial pressure of CO_2_ (levels of *p*CO_2_ in seawater in μatm), carbonate ions concentration (CO_3_^2-^ in μmol Kg^-1^), saturation state of the seawater with respect to aragonite minerals (Ω_arag_).

| CO_2_ System parameters | Experimental treatments (nominal levels of CO_2_ ppm) | |
| --- | --- | --- |
|  | 400 (current) | 1000 (year 2100) |
| pH in situ (pH units) | 7.99± 0.06 | 7.65± 0.05 |
| Salinity (psu) | 29.40± 0.65 | 29.26± 0.61 |
| Temperature in situ (°C) | 11.70± 0.67 | 11.08± 0.45 |
| TA (μmol Kg^-1^) | 2000.77± 172.46 | 2068.50± 182.90 |
| *p*CO_2_ in situ (μatm) | 414.06± 97.53 | 1005.15± 183.99 |
| [CO_3_^2-^] in situ (μmol Kg^-1^) | 97.54± 5.39 | 49.09± 3.70 |
| Ω_calc_ | 2.40± 0.13 | 1.21± 0.09 |
| Ω_arag_ | 1.51± 0.08 | 0.76± 0.06 |

**Supplementary Table 6:** Key applications of choice experiments applied to consumer preference in different product and food attributes.

| STUDY | CATEGORY | SPECIFIC | WELLINGNESS TO PAY FOR.. | Nº SURVEY |
| --- | --- | --- | --- | --- |
|  |  |  |  |  |
| Xu et al., 2012 | Seafood | Seafood | Eco-labeled seafood | 366 |
| Brécard et al., 2012 |  |  | Ecological label | 911 |
| Afnes et al., 2006 |  | Fish | Salmon color | 115 |
| Anderson, 2001 |  |  | Salmon color | − |
| Cardoso et al., 2013 |  |  | Seafood preferences, consumption frequencies | 1083 |
| Shyam, 2013 |  |  | High value fishes | 540 |
| Alló and Loureiro, 2017 |  |  | Salmon (type of production) | 115 |
| Olesen et al., 2010 |  |  | Salmon (type of production) | 115 |
| Batzios et al., 2003a |  | Shellfish | Basic marketing aspects (i.e. hygiene certification, media influence, etc) | 400 |
| Batzios et al., 2004 |  |  | Marketing and quality/safety | 409 |
| Lusk y Schroeder, 2004a | Food | Beef | Types of beef | 114 |
| Van Loo et al., 2011 |  | Chicken | General organic label or certified | 976 |

**Supplementary Table 7: Example of choice set.** This scenario considered, consumers with two different mussels profiles, with changes in attributes. In each choice scenario, a consumer was able to select one of the profiles, or the “opt-out” (none of these) alternative. Each consumer was presented with six choice situations randomly assigned.

|  | **OPTION 1** | **OPTION 2** | **NONE** |
| --- | --- | --- | --- |
| **Shell Size** | Small (5cm.) | Large (7cm.) | - |
| **Nutritional Quality** | High | Low | - |
| **Shell color**  **(At harvest)** | 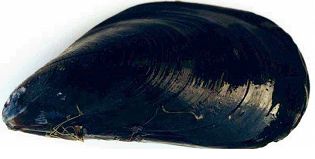 | 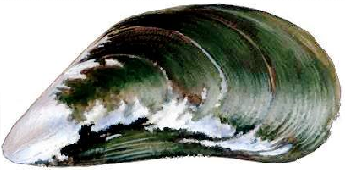 | - |
| **Meat Color** | 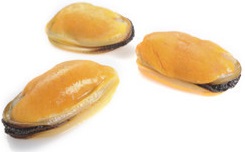 | 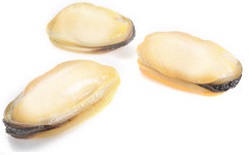 | - |
| **Price**  **(250g. meat)** | $1.700 (US$2.8) | $1.000 (US$1.6) | $0 |

The objective was testing if consumers have preferences for differences on meat color. We relay on evidence showing that consumer does have preferences related to meat color for salmon (Alfnes et al., 2006). Thus, within the survey we proposed different scenarios to test this hypothesis.
